# Significance of Image Guidance to Clinical Outcomes for Localized Prostate Cancer

**DOI:** 10.1155/2014/860639

**Published:** 2014-07-13

**Authors:** Qiuzi Zhong, Hong Gao, Gaofeng Li, Xia Xiu, Qinhong Wu, Ming Li, Yonggang Xu

**Affiliations:** Department of Radiotherapy, Beijing Hospital, No. 1 Dongdan Dahua Road, Dongcheng District, Beijing 100730, China

## Abstract

*Purpose*. To compare toxicity profiles and biochemical tumor control outcomes between patients treated with image-guided intensity-modulated radiotherapy (IG-IMRT) and non-IGRT intensity-modulated radiotherapy (IMRT) for clinically localized prostate cancer. *Materials and Methods*. Between 2009 and 2012, 65 patients with localized prostate cancer were treated with IG-IMRT. This group of patients was retrospectively compared with a similar cohort of 62 patients who were treated between 2004 and 2009 with IMRT to the same dose without image guidance. *Results*. The median follow-up time was 4.8 years. The rectal volume receiving ≥40 and ≥70 Gy was significantly lower in the IG-IMRT group. Grade 2 and higher acute and late GI and GU toxicity rates were lower in IG-IMRT group, but there was no statistical difference. No significant improvement in biochemical control at 5 years was observed in two groups. In a Cox regression analysis identifying predictors for PSA relapse-free survival, only preradiotherapy PSA was significantly associated with biochemical control; IG-IMRT was not a statistically significant indicator. *Conclusions*. The use of image guidance in the radiation of prostate cancer at our institute did not show significant reduction in the rates of GI and GU toxicity and did not improve the biochemical control compared with IMRT.

## 1. Introduction

The practice of primary external-beam radiotherapy (EBRT) for prostate cancer has changed dramatically. There were many studies showing intensity-modulated radiotherapy (IMRT) that improved survival rates and reduced side effects comparing with conventional three-dimensional conformal radiation therapy (3D-CRT) for the treatment of prostate cancer [1, 2]. With clinically proven benefit, IMRT became the standard care for prostate cancer. Image-guided radiotherapy (IGRT) uses 3D volumetric image to position the patient to treatment position before radiation delivery. IGRT potentially represents a more accurate form of dose delivery for patients receiving radiotherapy for prostate cancer. Given the highly conformal nature of this form of therapy, surrounding normal tissues such as the rectum and bladder can be effectively spared from being exposed to high radiation doses, leading to fewer treatment complications [3, 4]. However, due to the longer treatment time and additional cost, it is important to understand the limitation of these new technologies and determine whether a dosimetric gain can translate into clinical benefit.

Since 2009, IG-IMRT has been the standard treatment at our institution for all prostate cancer patients. In this study, we retrospectively compared the outcomes of a cohort of patients treated with definitive IG-IMRT to the outcomes in a cohort treated with the same dose with IMRT but without image-guided position correction. The toxicity and tumor-control outcomes of these two patient cohorts were compared.

## 2. Methods and Materials

### 2.1. Patient Characteristics

From 2009 to 2012, 65 patients were treated with IG-IMRT to a dose of 76–80 Gy for stages T1–T3 prostate cancer. This group of patients was retrospectively compared with a similar cohort of 62 patients who were treated between 2004 and 2008 with IMRT to the same prescription dose, but without cone beam computed tomography (CBCT) guidance. The clinical characteristics of these two patient populations are shown in Table 1. Patients were staged according to the 2005 American Joint Committee on Cancer staging classification system. All patients had biopsy-proven adenocarcinoma that was classified according to the Gleason grading system, with neoadjuvant and concurrent androgen suppression therapy for prostate cancer.

For patients who received neoadjuvant androgen deprivation therapy (ADT; *n* = 108; 85.04%), the median neoadjuvant ADT time was 8 months. The median preradiotherapy prostate-specific antigen (PSA) was 0.68 ng/mL.

### 2.2. Target Definition

All patients underwent CT simulation approximately 2–4 days before starting treatment. A 5 mm slice thickness was used for CT image acquisition. The patients were instructed to have a full bladder and empty rectum at CT simulation and for each fraction of delivery.

Eclipse (Varian Medical Systems, Palo Alto, CA) was used for treatment planning. The treatment techniques, prescription dose, and dose-volume constraints for the target and normal tissues used for both patient groups were the same for the initial 66–70 Gy. For both groups, the planning target volume (PTV) included the prostate and entire seminal vesicles with a 8–10 mm margin except at the prostate-rectal interface, where a 6 mm margin was used. For the IG-IMRT group, plan was modified for the last 5 fractions (10 Gy) with reduced PTV volume. In that boost plan, clinical target volume (CTV) was expanded with a three-dimensional margin of 3-4 mm to create a smaller PTV for planning.

The rationale at that time was not to deviate too much from our previous IMRT technique. But we hypothesized that the use of daily IGRT during the boost phase should justify reduced margin, given its enhanced positioning accuracy.

The pelvic lymph nodes were irradiated in patients with an estimated risk of pelvic nodal involvement of ≥15% using the Roach formula [5]. The pelvic nodal regions included the internal and external iliac, common iliac, perirectal and presacral nodal regions [6], and common iliac lymph nodes up to L5/S1.

The bladder, rectum, small bowel, and femoral heads were delineated as OARs. The rectum volume included the entire rectal wall and the lumen, extending from the anus to the rectosigmoid flexure. The bladder was contoured from its base to the dome.

### 2.3. Treatment Planning

#### 2.3.1. IMRT Group

Single-fraction dose of 2 Gy was prescribed to PTV. If CTV included pelvic nodal regions, the IMRT plan was given in two phases. In the first phase, the PTV1 which included pelvic lymphatic drainage area, seminal vesicles, and prostate received 48–50 Gy in 24-25 fractions. Then a second CT simulation was performed. The PTV2 which only included prostate and seminal vesicles received 28–30 Gy boost in 14-15 fractions to a total PTV prostate dose of 76–80 Gy in 38–40 fractions. All doses were prescribed to a minimum isodose line encompassing ≥95% of the PTV. Patients were treated with 7 fields IMRT using Varian linear accelerators iX (Varian Medical Systems, Palo Alto, CA) equipped with a 120-leaf Varian millennium multileaf collimators. If CTV only included the prostate and entire seminal vesicles, the IMRT plan was finished in one phase: PTV received 76–78 Gy in 38–40 fractions. Doses to OARs were limited below thresholds. The dose constraints were rectum volume: *V*_70_ < 25% and *V*_50_ < 50%; bladder: *V*_65_ < 30% and *V*_50_ < 50%; and femur: *V*_50_ < 5%.

#### 2.3.2. IG-IMRT Group

The prescription dose was the same with IMRT group. The only difference was in the last 5 fractions where a new plan was generated with reduced PTV margins.

### 2.4. Treatment Delivery and Outcomes Analyses

In the IG-IMRT cohort, image guidance was achieved by using the onboard imaging (OBI) system on the Varian iX linear accelerator. Planning CT and the verification CBCT were registered first with automatic bony registration, followed by a manual registration based on the soft-tissue alignment and prostate position in the CBCT. No prostate-implanted markers were used. Daily imaging was performed during the first three fractions. Thereafter, IGRT was utilized one to two times weekly. For the last five fractions of treatment, CBCT was performed everyday. Please note that most of studies reported on prostate cancer using IGRT involved daily CBCT [7–9]. In our center, for the purpose of saving treatment time and minimizing additional radiation exposure, CBCT was only used 10–15 times through the entire course of treatment. The average number of CBCT was 12 times per patient. Positioning errors were corrected online prior to treatment every time a CBCT was acquired.

Dose-volume histogram endpoints were retrospectively collected for all 127 patients. Using the composite plans, the bladder volume and rectum volume receiving ≥40 (*V*_40_), ≥50 (*V*_60_), ≥60 (*V*_60_), and ≥70 (*V*_70_) Gy of the target volume were recorded. The assessment of toxicity was performed every week during treatment. Follow-up evaluations after RT were performed at intervals of 3 to 6 months, and the median follow-up time was 4.8 years (range, 1–8 years). The median follow-up intervals for the IMRT and IG-IMRT cohorts were 70 and 23 months, respectively. PSA relapse was defined according to the Phoenix definition (absolute nadir plus 2 ng/mL dated at the call). All statistical analyses were performed using Statistical Package for Social Sciences, version 16.0 (SPSS, Chicago, IL). Gastrointestinal (GI) and genitourinary (GU) toxicities were scored using the common terminology criteria for adverse events (CTCAE), version 3.0, grading schemes. Results were considered significant at a *P* value <0.05.

## 3. Results

The patients' characteristics are summarized in Table 1. All patients completed RT, with no treatment breaks. The planning target volume dose coverage was not significantly different between IMRT and IG-IMRT for the prostate, seminal vesicles, and lymph nodes (*P* > 0.05).

### 3.1. Dosimetric Avoidance of Organs at Risk

Table 2 summarized the differences in dosimetry of the bladder and rectum for IMRT and IG-IMRT groups. The volume of bladder receiving ≥40, ≥50, ≥60, and ≥70 Gy was not significantly different from two groups (*P* > 0.05). The rectal volume receiving ≥40 (61.3 versus 53.4 Gy; *P* = 0.022) and ≥70 Gy (18.7 versus 14.5 Gy; *P* = 0.006) was significantly lower at the IG-IMRT group.

### 3.2. Acute Toxicity

Regarding the acute toxicity, 77 patients (67%) experienced grade 1 symptoms and 27 (21%) experienced grade 2 toxicity. No grade 3 or higher acute events were observed. The most common GU and GI radiation toxicities were frequency/urgency and diarrhea.  Table 3 compared the incidence of acute rectal and urinary toxicities observed in the IG-IMRT and IMRT treatment groups. No differences were observed between the treatment groups for grade 2 and higher acute GI and GU toxicity rates (*P* = 0.986; *P* = 0.6).

### 3.3. Late Toxicity

The grade 2 or higher late rectal toxicity was similar in and low for both the IGRT and non-IGRT groups (1.5% and 3.2%, resp.; *P* = 0.967). The most common late GI radiation effect was mild bleeding. One patient required minor cauterization for hemostasis. No late toxicity grade 4 was observed. The incidence of grade 0-1 and 2 GU toxicity was 96.8% and 3.2%, respectively, with IMRT compared with 98.5% and 1.5%, respectively, with IG-IMRT (Table 4). In a logistic regression analysis, IGRT was not associated with significantly less acute and late toxicity compared with non-IGRT patients (*P* > 0.05).

### 3.4. Biochemical Control

**Figure 1:**
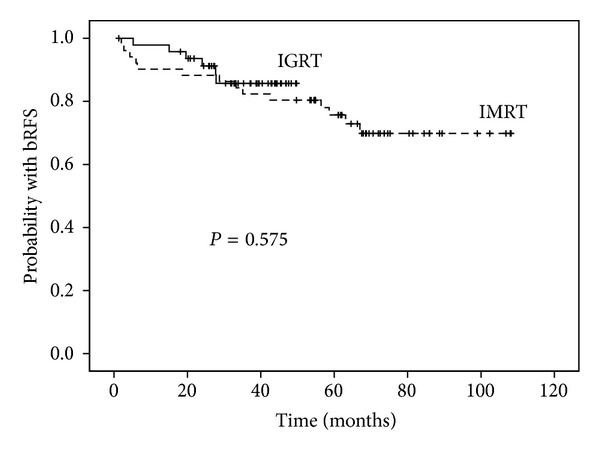
Comparison of prostate-specific antigen relapse-free survival outcomes between the high-risk patients treated with image-guided radiotherapy (IG-IMRT) and those treated with IMRT alone.

No significant improvement in biochemical control at 5 years was observed in the low- and intermediate-risk patients in IG-IMRT cohort compared with IMRT group (*P* = 0.427). As noted in Figure 1, the same result was observed in the high-risk group patients (*P* = 0.575). In a Cox regression analysis identifying predictors for PSA relapse-free survival, only preradiotherapy PSA was significantly associated with biochemical control; image guidance was not (Table 5).

## 4. Discussion

Intuitively, IG-IMRT would provide enhanced delivery accuracy than IMRT alone. Thus IG-IMRT by default should demonstrate reduction in normal organ toxicity and better tumor-control outcomes. Chung et al. [10] have reported reduced acute bladder and rectal toxicities associated with whole pelvic IGRT compared with IMRT in prostate cancer patients. Their study used smaller PTV margins for IG-IMRT group attributing it to the enhanced accuracy of image guidance. In 2009, de Crevoisier et al. described a series of 107 patients who underwent IG-IMRT (CBCT used in 67% of patients, fiducial markers in 28%, and ultrasounds in 5%), with a median total dose of 76 Gy [11]. Grades 2 and 3 acute rectal toxicity rates were only 7% and 0%, respectively. More recently, a retrospective comparison by Zelefsky et al. [12] noted that IGRT was associated with an improvement in biochemical tumor control among high-risk patients and a lower rate of late urinary toxicity compared with high-dose IMRT (86.4 Gy).

However, there are many factors that can potentially compromise the benefit of image guidance. For that reason, although numerous studies have demonstrated the dosimetric superiority of IG-IMRT compared with IMRT, very limited numbers of studies have shown that these dosimetric differences can lead to tangible differences to the patient in terms of toxicity and tumor control [12–14]. Our study intended to show that without carefully cultivated implementation plan, addition of image guidance may not translate to clinically significant outcome improvements. In our study, both groups were treated to the same radiation dose level and with similar margins for the PTV. The patients' characters of two groups were almost identical (*P* > 0.05). Our results showed that there were no significant differences in side effects and clinical control between IG-IMRT and IMRT groups. Although these results are a little bit unexpected, they are not surprising. We believe the following factors could potentially compromise the benefit of image guidance.

*(i) Frequency of Image Guidance*. Most studies that demonstrated significant IGRT benefit used daily image guidance. Han et al. reported that daily image guidance was needed throughout the course of treatment in conformal radiotherapy for esophageal cancer. Even if the most frequent less-than-daily IG strategy was to be used, substantial residual setup errors would occur for treatment fractions without image guidance, which could lead to significant daily dose variations for the target volume and adjacent normal tissues. However, one of the downsides of daily IGRT is the extra radiation dose it introduces [15–17].  Table 6 provides a list of the doses per fraction associated with a list of different IGRT modalities. There was at least one report stipulating that the extra imaging dose was associated with more toxicity if not computed in the total dose delivered [18]. Another consideration is the cost and resources. A randomized trial in France showed that the average increase of cost for CBCT-based daily imaging guidance was 43% higher than weekly imaging. They suggested that daily IGRT combined with intensity-modulated RT (IMRT) should only be considered in high-dose radiation delivery situations [19]. For the sake of reducing cost and imaging dose, we designed our study with less than daily image guidance. For each prostate patient treatment course, only 10–16 (mean = 12) CBCTs were performed. For a treatment extending over a large number of fractions, the impact of day-to-day delivery variation, or random error, is usually less important than systematic errors [20–22]. However, when the number of fractions is reduced, then random error (amplified without image guidance) can have a larger negative impact [21]. Lack of daily CBCT in our study could be one of the reasons why the side effects of two groups were not significantly different.

*(ii) PTV Margins*. One challenge facing RT is organ motion. This is especially relevant for prostate cancer. Recent studies have shown a significant degree of prostate motion, both inter- and intrafraction, and its adverse effects on IMRT dosimetry [23–25]. Appropriate margins have to be applied to ensure the targets receiving desired prescription dose. The magnitude of margin depends on the delivery technique. Various strategies have been studied, such as implanting fiducial gold seed markers (FMs) into the prostate [26–29], implanting beacon transponders into the prostate that allows for continuous, real-time localization [30–32], using an endorectal balloon to limit prostate motion during treatment [33], verifying the prostate position using B-mode acquisition, and targeting transabdominal ultrasonography before each treatment [34–37] or helical tomotherapy [24, 38, 39] or with CBCT [40–42]. Collectively, these strategies represent different attempts at IGRT, ultimately permitting the reduction of the PTV margins because of improved setup accuracy and reproducibility. Only with reduced margin, IMRT planning can take full advantage of image guidance and generate plans with reduced normal organ dose. Otherwise, the treatment plan will be identical with or without image guidance. In our study, similar PTV margins were used in both groups.

Fiducial markers can facilitate the localization of prostate. Soft-tissue targeting (prostate) requires a higher-dose imaging technique, but high-contrast targets, such as bone or metallic fiducial markers, can be accurately visualized at imaging doses as low as 0.1 to 0.5 cGy. Different registration can result from which landmarks are used as the reference, either fiducial markers, soft-tissue registration, or bony anatomy structures. Alander et al. [43] investigated the prostate bed localization difference between soft-tissue registration and gold seed fiducial localization. They found that the gold seed fiducial localization is better than bone localization and soft-tissue registration in daily IGRT. The CBCT and the bony structure alignment can reduce setup errors as compared to the localization with skin marks. Ost et al. [44] used anterior rectal wall to match posterior wall of the prostate bed of the CBCT with the planning CT and the registration was performed by a radiation oncologist and a therapist. In our study, no fiducial markers were used. CBCT for first 2-3 treatment fractions and last five fractions were supervised by a radiation oncologist. Radiation therapists did the rest of registration sessions by bony alignment. If we had implanted fiducial markers, therapists alone would be able to perform the registration more consistently. This was the reason we kept the PTV margins similar between the two groups. Without margin reduction, the advantage of image guidance was not fully realized.

*(iii) Prescription Dose*. Total radiation dose delivered to the target was one of the most important predictors of long-term biochemical tumor control [45]. Published randomized trials [46–49] demonstrated significant improvements in biochemical tumor control for low-risk patients and intermediate- and high-risk patients with escalated dose. It is reasonable to assume that additional escalation of the radiation dose for intermediate-risk patients and, especially, high-risk patients would be associated with further improvements in tumor-control outcomes. Zelefsky et al. [45] reported 2551 patients with clinical stages T1–T3 prostate cancer. Dose levels of ≥75.6 Gy for low-risk patients were associated with improved long-term PSA-RFS outcomes, and for higher-risk patients they observed improved biochemical control with ≥81 Gy. In our study, the PTV dose of two groups was the same (76–80 Gy). So it was not a surprise to see that the clinical tumor-control outcome was similar between IG-IMRT and IMRT groups.

*(iv) Sample Size and Follow-Up Length*. As shown in Table 7, the percentage of grade 2 acute GI and GU toxicity was 17% and 42% in six previously reported studies. They were slightly higher than our IMRT and IG-IMRT groups. Statistically, there were no differences between our two groups due to our small sample sizes and the low probability of side effects. The incidence of grade 2 and higher late rectal and bladder toxicity was so low for both treatment groups (1.5% and 3%, resp.; *P* = 0.41) that only 1 or 2 patients had any problems. More patients would be required to demonstrate any difference in the tolerance profile of treated patients. In addition, the follow-up length in our study was relatively short with only 23 months (median) in the IG-IMRT group. Longer followup can potentially magnify the differences between the groups.

In conclusion, our study did not show significant improvement in biochemical control at 5 years in the low-, intermediate-, and high-risk prostate patients with IG-IMRT compared with IMRT alone; neither did we observe any statistical difference in grade 2 and higher acute GI and GU toxicity rates. We believe it was due to multiple factors including the frequency of image guidance, PTV margin and dose, sample size, and length of followup. In order to maximize the benefit of IGRT, those parameters should be carefully evaluated.

## Figures and Tables

**Table 1:** Patient characteristic.

Patient demographics	IG-IMRT (*n* = 65)		IMRT (*n* = 62)		*P* value
	*n*	%	*n*	%	
Preradiotherapy PSA					
<10	58	89.23	49	79.03	0.286
10–20	4	6.15	7	11.29	
>20	3	4.62	6	9.68	
Total Gleason score					
<7	10	15.38	12	19.35	0.559
7	20	30.77	23	37.10	
>7	34	52.31	27	43.55	
T stage					
T1	0	0	2	3.22	0.529
T2	30	46.15	27	43.55	
T3	35	53.85	33	53.23	
Age (y)					
<70	14	21.54	18	29.03	0.331
≥70	51	78.46	44	70.97	
NCCN risk					
Low	2	3.08	2	3.23	0.933
Intermediate	13	20.00	10	16.13	
High	50	76.92	50	80.64	
Neoadjuvant ADT					
Yes	56	86.15	52	83.87	0.718
No	9	13.85	10	16.13	

ADT: androgen deprivation therapy; NCCN: National Comprehensive Cancer Network; PSA: prostate-specific antigen.

**Table 2:** Comparison of doses received by organs at risk between IMRT and IG-IMRT groups.

	IG-IMRT	IMRT	*P*
Bladder			
*V*_40_ (%)	57.88	58.32	0.937
*V*_50_ (%)	41.17	42.28	0.796
*V*_60_ (%)	27.48	30.62	0.905
*V*_70_ (%)	17.09	21.01	0.782
Volume	180.46	163.33	0.598
Rectum			
*V*_40_ (%)	53.358	61.286	0.022
*V*_50_ (%)	39.945	40.131	0.949
*V*_60_ (%)	29.316	26.500	0.210
*V*_70_ (%)	14.493	18.745	0.006
Volume	60.74	73.35	0.200

*V*_40_, *V*_50_, *V*_60_, and *V*_70_ are the percentage of volume receiving ≥40, ≥50, ≥60, and ≥70 Gy, respectively. Data are presented as mean values.

**Table 3:** Comparison of acute rectal and urinary toxicities between patients treated with IG-IMRT and IMRT.

Acute toxicities	IG-IMRT (*n* = 65)		IMRT (*n* = 62)		Total (*n* = 127)	
	*n*	%	*n*	%	*n*	%
Rectal						
0	47	72.3	45	72.6	92	72.4
1	16	24.6	14	22.6	30	23.6
2	2	3.1	3	4.8	5	4.0
3	0	0.0	0	0.0	0	0.0
Urinary						
0	32	49.2	26	41.9	58	45.7
1	23	35.4	24	38.7	47	37.0
2	10	15.4	12	19.4	22	17.3
3	0	0.0	0	0.0	0	0.0

**Table 4:** Comparison of late rectal and urinary toxicities between patients treated with IG-IMRT and IMRT.

Late toxicities	IG-IMRT (*n* = 65)		IMRT (*n* = 62)		Total (*n* = 127)	
	*n*	%	*n*	%	*n*	%
Rectal						
0	60	92.3	57	92.0	117	92.1
1	4	6.2	3	4.8	7	5.5
2	1	1.5	1	1.6	2	1.6
3	0	0.0	1	1.6	1	0.8
Urinary						
0	60	92.3	54	87.1	114	89.7
1	4	6.2	6	9.7	10	7.9
2	1	1.5	2	3.2	3	2.4
3	0	0.0	0	0.0	0	0.0

**Table 5:** Cox regression analysis for predictors identifying PSA relapse-free survival.

Cox regression	*B*	SE	*P*	Exp(*B*)	95% CI	
					Lower	Upper
Gleason score ≥8	−0.120	0.480	0.802	0.887	0.346	2.273
T stage	0.419	0.460	0.362	1.520	0.617	3.744
Age	0.012	0.024	0.609	1.012	0.996	1.062
Preradiotherapy PSA	0.019	0.004	0.000	1.019	1.011	1.028
IG	0.609	0.546	0.265	1.838	0.630	5.362

*B*: regression coefficient estimate; SE: standard error; CI: confidence interval; IG: image guidance; PSA: prostate-specific antigen.

**Table 6:** Added dose and time per modality per fraction in pelvis IGRT [16, 50–53].

Modality	Dose at midbody (cGy)	Time (min)∗	Available examples^&^
Ultrasound	0	2-3	BATCAM, Clarity
Plain kV^†^	0.1–0.6	0.1–3	Cyberknife, ExacTrac
Plain MV^†^	1–10	0.1–3	Various EPID and portal devices
kV CBCT^‡^	2-3	2–4	ARTISTE, OBI, XVI
MV CBCT	5–15	2-3	MVision
kV FBCT^§^	0.8–2.8	15	CTVision, EXaCT
MV FBCT	1.5–3	2-3	Tomotherapy

FBCT: fan-beam computed tomography scanning.

*excludes image interpretation and action on observations.

^&^BATCAM, Best nomos, Pittsburgh, PA; Clarity and XVI, Elekta, Stockholm, Sweden; Cyberknife and Tomotherapy, Accuray, Sunnyvale, CA; EXaCT, ExacTrac, and OBI, Varian Medical Systems, Inc., Palo Alto, CA; ARTISTE, CTVision, and MVision, Siemens AG, Erlangen, Germany.

^†^For 2 incidences.

^‡^Full soft-tissue scan, 360°.

^§^involves couch rotation and CT translation because CT scanning is not on linac gantry.

**Table 7:** Acute gastrointestinal (GI) and genitourinary (GU) toxicity in the literature on radiation therapy of prostate cancer.

Study	Patients (*n*)	Total dose (Gy)	Fractions (*n*)	EQD2 (Gy)	IGRT	Acute GI (%)		Acute GU (%)	
						Grade 2	Grade 3	Grade 2	Grade 3
Pollack et al. 2006 [7]	100	76 (D95) 70.4 (D95)	38 28	76 84.6	Daily	8 18	0 0	54 40	2 8
Fonteyne et al. 2008 [8]	230	78 (median)	38	79.1	Daily	11	0	41	7
Ghadjar et al. 2008 [54]	39	80 (median)	40	80	Daily	3	0	56	8
Lips et al. 2008 [9]	331	76 (mean)	35	79.7	Daily	30	0	47	3
Marchand et al. 2010 [55]	55	72.2	38	70.1	Daily	12.7	0	38.2	1.8
Deville et al. 2010 [56]	30	79.2	44	74.7	Daily	13	0	50	0
Our own data	127	78.6 (mean)	39	78.9	Customized	4	0	17.3	0
